# Germline *GPR161* Mutations Predispose to Pediatric Medulloblastoma

**DOI:** 10.1200/JCO.19.00577

**Published:** 2019-10-14

**Authors:** Matthias Begemann, Sebastian M. Waszak, Giles W. Robinson, Natalie Jäger, Tanvi Sharma, Cordula Knopp, Florian Kraft, Olga Moser, Martin Mynarek, Lea Guerrini-Rousseau, Laurence Brugieres, Pascale Varlet, Torsten Pietsch, Daniel C. Bowers, Murali Chintagumpala, Felix Sahm, Jan O. Korbel, Stefan Rutkowski, Thomas Eggermann, Amar Gajjar, Paul Northcott, Miriam Elbracht, Stefan M. Pfister, Udo Kontny, Ingo Kurth

**Affiliations:** ^1^ RWTH Aachen University, Aachen, Germany; ^2^ European Molecular Biology Laboratory, Heidelberg, Germany; ^3^ St Jude Children’s Research Hospital, Memphis, TN; ^4^ Hopp Children’s Cancer Center Heidelberg, Heidelberg, Germany; ^5^ German Cancer Research Centre, Heidelberg, Germany; ^6^ University Medical Center Hamburg-Eppendorf, Hamburg, Germany; ^7^ Gustave Roussy Cancer Center, Villejuif, Paris, France; ^8^ Université Paris Descartes, Paris, France; ^9^ University of Bonn, Bonn, Germany; ^10^ University of Texas Southwestern Medical School, Dallas, TX; ^11^ Baylor College of Medicine, Houston, TX; ^12^ University Hospital Heidelberg, Heidelberg, Germany

## Abstract

**PURPOSE:**

The identification of a heritable tumor predisposition often leads to changes in management and increased surveillance of individuals who are at risk; however, for many rare entities, our knowledge of heritable predisposition is incomplete.

**METHODS:**

Families with childhood medulloblastoma, one of the most prevalent childhood malignant brain tumors, were investigated to identify predisposing germline mutations. Initial findings were extended to genomes and epigenomes of 1,044 medulloblastoma cases from international multicenter cohorts, including retrospective and prospective clinical studies and patient series.

**RESULTS:**

We identified heterozygous germline mutations in the G protein-coupled receptor 161 (*GPR161*) gene in six patients with infant-onset medulloblastoma (median age, 1.5 years). *GPR161* mutations were exclusively associated with the sonic hedgehog medulloblastoma (MB_SHH_) subgroup and accounted for 5% of infant MB_SHH_ cases in our cohorts. Molecular tumor profiling revealed a loss of heterozygosity at *GPR161* in all affected MB_SHH_ tumors, atypical somatic copy number landscapes, and no additional somatic driver events. Analysis of 226 MB_SHH_ tumors revealed somatic copy-neutral loss of heterozygosity of chromosome 1q as the hallmark characteristic of *GPR161* deficiency and the primary mechanism for biallelic inactivation of *GPR161* in affected MB_SHH_ tumors.

**CONCLUSION:**

Here, we describe a novel brain tumor predisposition syndrome that is caused by germline *GPR161* mutations and characterized by MB_SHH_ in infants. Additional studies are needed to identify a potential broader tumor spectrum associated with germline *GPR161* mutations.

## INTRODUCTION

Cancer predisposition syndromes are defined by germline mutations that result in a highly or moderately increased tumor risk in affected individuals. Knowledge of these syndromes has a profound impact on patient care and the prevention of malignant disease through the provision of genetic counseling and careful surveillance of families who are at risk.^1^ Many of the genes mutated in cancer predisposition syndromes are also somatically mutated in sporadic types of cancer. Thus, understanding the molecular function of these genes can lead to new therapeutic concepts in both sporadic and heritable tumors. In children and adolescents with cancer, tumor predisposition as a result of germline mutations is found in at least 7% to 10%.^2-4^ Medulloblastoma (MB), a tumor that originates in the cerebellum and dorsal brainstem, has a peak incidence in childhood and makes up a large proportion of embryonal brain tumors. Consensus molecular subgroups of MB include WNT, sonic hedgehog (SHH), group 3, and group 4, each showing distinct transcriptional and epigenetic profiles.^5^ Recent advances in deep molecular phenotyping suggest additional MB subtypes with distinct somatic driver mutations and epigenetic signatures.^6-8^ The SHH subgroup (MB_SHH_) is characterized by a constitutive transcriptional and genomic activation of the SHH pathway^9^; driver mutations in *PTCH1*, *SUFU, SMO*, *TERT, DDX3X, TP53,* and *KMT2D*; and structural aberrations that affect most frequently chromosomes 9, 10, and 17. Most MBs are thought to develop sporadically, but inherited forms also exist, most often in children with MB_SHH_.^4^ Heritable predisposition to MB is observed in Li-Fraumeni syndrome,^10^ *APC*-associated polyposis,^11^ subtypes of Fanconi anemia,^12,13^ and Gorlin syndrome (nevoid basal-cell carcinoma syndrome). The latter is associated with germline mutations in *SUFU*^14-16^ and *PTCH1*,^17,18^ which play crucial roles in the SHH pathway. Active SHH signaling is a key element of embryonic development and cell differentiation.^19^ Here, we describe the SHH regulator G protein-coupled receptor 161 (*GPR161*) as a novel brain tumor predisposition gene in children.

## METHODS

### Patients

Written informed consent was obtained from study participants after approval from the institutional review boards at the participating institutions (Uniklinik RWTH Aachen: EK302-16). Consent was obtained according to the Declaration of Helsinki. The study had access to international multicenter MB studies, including retrospective cohorts (International Cancer Genome Consortium [ICGC] PedBrain, Medulloblastoma Advanced Genomics International Consortium, CEFALO series, and clinic of pediatric oncogenetics from Gustave Roussy), and prospective cohorts from clinical studies or patient series (SJMB03, SJMB12, SJYC07, and I-HIT-MED).^4,20^

### Germline and Tumor Sequencing

Whole-exome sequencing (WES) of the index patient (M20769) was done at the Institute of Human Genetics, Uniklinik RWTH Aachen, using the Nextera Rapid Capture Exome kit (version 1.2; Illumina, San Diego, CA). The library was sequenced on a NextSeq500 Sequencer with 2 × 75 cycles on a high-output flow cell. For sample MB11_06, WES data for germline and tumor samples were generated at the German Cancer Research Centre (Heidelberg, Germany) using Agilent SureSelect Human All Exon V5 (Agilent Technologies, Santa Clara, CA) without untranslated regions and sequenced on an Illumina HiSEq 4000 System (paired-end mode). For sample MB13_03, whole-genome sequencing data for germline and tumor samples were generated at the German Cancer Research Centre and sequenced on the Illumina HiSeq X Ten System (paired-end mode). For samples SJMB335, SJMB303, and SJMB054, WES data for germline and tumor samples were generated at St Jude Children’s Research Hospital (Memphis, TN) using the Agilent SureSelect Human All Exon V5 kit. For the detection of single-nucleotide variations (SNVs), small insertions and deletions (indels), fusions, and copy number aberrations, the customized enrichment/hybrid capture–based next-generation sequencing gene panel analysis covering 130 genes of particular relevance in brain tumors was applied, as previously described.^21^

### Somatic and Germline Variant Calling

Sequencing data were processed using a standardized alignment and somatic variant calling pipeline, which was developed in the context of the ICGC Pan-Cancer Analysis of Whole Genomes (PCAWG) project (https://dockstore.org/containers/quay.io/pancancer/pcawg-dkfz-workflow). In brief, reads were aligned to the phase II reference human genome assembly of the 1000 Genomes Project, including decoy sequences (hs37d5), using BWA-MEM (version 0.7.15). Somatic SNVs were called with the previously described SAMtools-based German Cancer Research Centre pipeline adjusted for ICGC PCAWG settings, and short somatic indels were called using Platypus.^22,23^ The CNVkit library^24^ and ACEseq^25^ were used to infer and visualize somatic genome copy number from the WES and whole-genome sequencing data. Loss of heterozygosity (LOH) analysis for tumor-only panel sequencing data was based on known germline variants (1000 Genomes Project) in target and off-target regions, 20× sequencing coverage, and one or more read support for the reference and alternative allele. Germline genomes and exomes were analyzed using the freebayes (version 1.1.0) pipeline with ICGC PCAWG settings^26^ and deleterious germline variants—SNVs, multi-nucleotide variants, indels, and complex variants—were inferred using automated pipelines and manually curated. For sequencing data of the index case, demultiplexing and fastq file generation were performed using bcl2fastq2 (version 2.2; Illumina) and read alignment and variant calling using the automated SeqMule pipeline (v1.2.6).^27^ For germline variant detection in the index patient, different variant callers were used (GATKLite UnifiedGenotyper, SAMtools, freebayes consensus) and variants shared by at least one pair of variant callers were written to the final variant file. Variant annotation and bioinformatics prioritization were performed using KGGSeq (version 1.0; 20 June 2018).^28^ Synonymous variants and variants with a minor allele frequency greater than 0.75% in public databases, that is, gnomAD, ExAC, 1000 Genomes Project, and National Heart, Lung, and Blood Institute Exome Sequencing Project, were excluded.

### DNA Methylation Array Processing

DNA from tumor tissue with tumor cell content greater than 80% by histopathologic evaluation was extracted from formalin-fixed, paraffin-embedded tissue using the automated Maxwell system with the Maxwell 16 formalin-fixed, paraffin-embedded Plus LEV DNA Purification Kit (Promega, Madison, WI). We performed DNA methylation profiling of all samples using the Infinium MethylationEPIC (850k) BeadChip (Illumina) or Infinium HumanMethylation450 (450k) BeadChip (Illumina) array, as previously described.^29^ Filtering and genome-wide copy number analyses were performed using the conumee package in R (http://www.bioconductor.org) as previously described.^29^

## RESULTS

**FIG 1.**
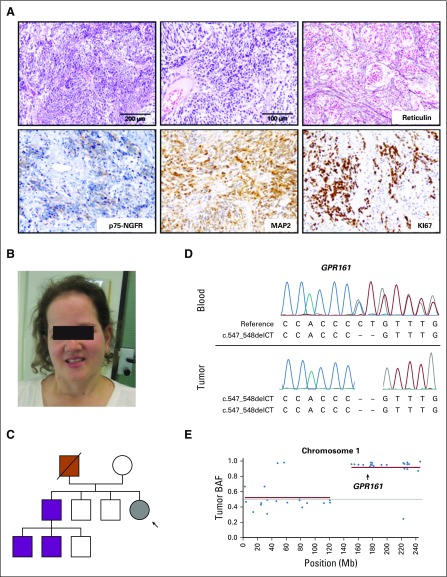
Index patient with medulloblastoma and germline *GPR161* mutation. (A) Desmoplastic/nodular medulloblastoma, SHH-activated, *TP53* wild type (WHO grade IV) in the index case (M20769). Highly cellular, undifferentiated small-cell neoplasm with increased mitotic activity. Silver impregnation shows an increased density of argyric fibers. The cells express the neural marker MAP2 and the SHH target protein p75-NGFR. (B) Index patient (27 years old). (C) Pedigree of the index patient (gray) with MB (black arrow). Father (red) was carrier of the *GPR161* germline mutation and died from colorectal cancer. Asymptomatic *GPR161* mutation carriers are indicated in purple. (D) Sanger sequencing-based validation of the germline *GPR161* frameshift mutation (c.547_548delCT) in peripheral blood (upper panel) and medulloblastoma (lower panel) of the index case. (E) Loss of heterozygosity analysis of chromosome 1 based on targeted gene panel sequencing of tumor DNA. *GPR161* location is highlighted with an arrow.

Our index patient was a female (M20769) with an extensive history of neoplasia that began with the diagnosis of MB at the age of 12 months. Histologic evaluation showed a highly cellular, undifferentiated small-cell neoplasm with increased mitotic activity. Cells had hyperchromatic nuclei and a scant cytoplasm. Silver impregnation demonstrated an increased density of argyric fibers and fiber-rich areas of ensheathed fiber-free islands of tumor cells. Cells expressed the neural marker MAP2 and the SHH target protein p75-NGFR (Fig 1A). Consistent with SHH activation, OTX2 was negative, and TP53 was not accumulated (not shown). DNA methylation profiling classified the tumor into the MB_SHH_ subgroup. According to the revised WHO classification of tumors of the CNS 2016, the tumor classifies as desmoplastic/nodular, SHH-activated, *TP53* wild-type MB (WHO grade IV).^30^ At age 16 years, the patient developed her first basal-cell carcinoma (BCC) and has subsequently gone on to develop 10 more BCCs, all within the radiation field and all amenable to surgical removal. By age 18, the patient underwent a total thyroidectomy for a multinodular goiter, and at 23 years old, the patient underwent the excision of a rectal tubular adenoma with low-grade dysplasia, a low-grade intraepithelial neoplasia in the stomach, and several hyperplastic serrated polyps. A meningioma in the right temporal region was removed at age 24. The patient, currently age 29 years, has a microcephaly and a mild frontal bossing and in summary fulfills some, but not all, criteria of Gorlin syndrome (Fig 1B).

**FIG 2.**
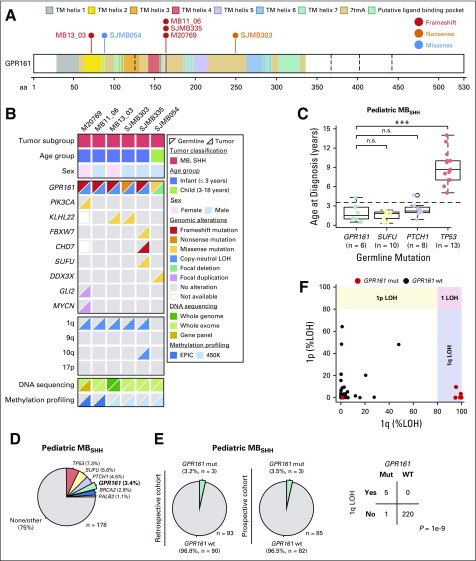
Genomic landscape of *GPR161*-associated medulloblastoma. (A) Germline *GPR161* mutations in patients with MB. (B) Demographic and molecular characteristics of *GPR161*-associated MB. (C) Age at diagnosis across pediatric patients with pathogenic germline mutations in MB_SHH_ predisposition genes. (D) Frequency of pathogenic germline mutations in pediatric MB_SHH_. (E) Frequency of germline *GPR161* mutations in retrospective and prospective pediatric MB_SHH_ cohorts. (F) Frequency of somatic 1p/1q LOH events in MB_SHH_ tumors and association between 1q LOH and *GPR161* mutation status. LOH, loss of heterozygosity; MB, medulloblastoma; Mut, mutant; SHH, sonic hedgehog; WT, wild type.

Matched WES was performed using DNA extracted from the patient’s MB tumor and blood. Analysis revealed no candidate germline mutation in consensus MB predisposition genes^4^; no somatic copy-number alterations affecting MB_SHH_ hallmark chromosomes 9, 10, and 17; and no somatic mutations in MB_SHH_ driver genes (eg, PTCH1, SUFU, TP53; Fig 2B). However, exome-wide analysis for rare damaging germline mutations revealed a frameshift mutation in *GPR161* on chromosome 1q24.2 (Figs 1D and 2A), and subsequent analysis of tumor DNA showed a somatic copy-neutral loss of heterozygosity (cnLOH) event on 1q (Fig 1E and Data Supplement). Of note, five additional rare germline protein-truncating variants were identified, but none of them were located in regions that showed focal deletions and/or LOH. Sanger sequencing confirmed that the 1q cnLOH event affected the wild-type *GPR161* allele and consequently led to somatic biallelic inactivation of *GPR161* (Fig 1D). These results provide the first clue that *GPR161* might be a novel MB predisposition gene.

To further corroborate the role of *GPR161* in predisposition to MB, we specifically analyzed the genomic data of 1,044 patients with MB who were enrolled in previous sequencing studies.^4,20^ These analyses revealed five additional patients with rare and damaging germline *GPR161* mutations (Figs 2A and 2B). Clinical details on these patients are summarized in the Data Supplement. Four patients were found to harbor *GPR161* protein-truncating variants and one patient was a carrier of a predicted damaging missense variant (SJMB054). Two patients (MB11_06 and SJMB335) shared the same frameshift mutation as that of the index patient; kinship analysis demonstrated that all three patients were unrelated.

Classification of 872 human MBs into four consensus MB subgroups^4,20^ revealed that all six patients with *GPR161* mutations developed MBs that belonged to the SHH subgroup (*P* < 5×10^−4^, Fishers’ exact test; Fig 2B). Moreover, all patients with *GPR161* mutations developed MB_SHH_ at a young age (median age, 1.5 years; *P* = .025, MWU test; Fig 2C), which is similar to the age at which patients with germline *PTCH1* and *SUFU* mutations develop MB_SHH_ (Fig 2C). Overall prevalence of germline *GPR161* mutations among pediatric (age < 18 years) and infant (age < 4 years) patients with MB_SHH_ was 3.4% (six of 178) and 5.5% (five of 91), respectively (Fig 2D). *GPR161* mutation carriers were also observed at a similar frequency in retrospective (3.2%; three of 93) and prospective (3.5%; three of 85) pediatric MB_SHH_ cohorts (Fig 2E). Of note, the frequency of germline mutations in *GPR161* (3.4%), *PTCH1* (4.5%), and *SUFU* (5.6%) was comparable among pediatric patients with MB_SHH_.

The latest edition of the WHO CNS tumor classification divides MB_SHH_ into *TP53* mutated and wild type, respectively.^30,31^ We assessed *TP53* mutation status across all MB_SHH_ and observed that all *GPR161*-associated MB_SHH_ were *TP53* wild type and represented 4.1% (six of 147) of pediatric patients with MB_SHH,_ *_TP53-wt_*. Recently, MB_SHH_ tumors have been further classified into four molecular subtypes.^8^ These include two infant subtypes SHH-γ (low risk) and SHH-β (high risk), which are also equivalent to infant-SHH-I and infant-SHH-II in another study.^32^ Classification of these infant *GPR161*-associated MB_SHH_ tumors into molecular subtypes demonstrated that the majority (five of six) belonged to the MB_SHH-β_ subtype; however, this was not statistically significant when considering all infant patients with MB_SHH_ in our series (*P* = .09). Finally, germline *GPR161* protein-truncating variants were significantly enriched in pediatric patients with MB_SHH_ compared with the general population (one in 30 patients *v* one in 1,664 controls in gnomAD; *P* = 2×10^−9^, Fisher’s exact test).

Analysis of tumor DNA in *GPR161* germline mutation carriers revealed LOH at the *GPR161* locus, unexceptionally leading to loss of the wild-type allele in all affected patients (*P* = .03, n = 6, binomial test; Fig 2B and Data Supplement). Other than this, no shared somatic driver event was detected, which further substantiates a role for *GPR161* deficiency as a driver event for MB development. Of note, patient SJMB054 had a somatic chromosome 6 monosomy, an alteration associated with MB_WNT_; however, a somatic *CTNNB1* mutation was not detected and the brain tumor methylation classifier score was highest for MB_SHH_ (MB_SHH_:0.9897 *v* MB_WNT_:0.0003). Patient SJMB335 had a somatic *SUFU* missense mutation, but clonal evolution analysis did not provide evidence for an *SUFU-*associated tumor as a result of low mutation frequency. Finally, the MB of the index patient showed low-level copy number gain at the *GLI2* locus, but this was not a true gene amplification and the tumor lacked a loss of 17p and a *TP53* mutation that usually accompanies a prototypical MB with *GLI2* amplification. In summary, biallelic *GPR161* inactivation occurred in all six patients with MB that otherwise did not harbor any other driver events that affect the SHH pathway (Fig 2B).

Remarkably, five of six patients with MB demonstrated an inactivation of *GPR161* as a result of cnLOH of 1q and in one tumor a focal approximate 425-kb deletion spanned all exons of *GPR161*. Recurrent cnLOH of 1q so far has not been reported in the MB literature and we therefore assessed the overall frequency of 1q cnLOH events among pediatric patients with MB_SHH_ and its association with *GPR161* mutation status (Fig 2B). This analysis revealed that 1q cnLOH is exclusively present in tumors with germline *GPR161* mutations (*GPR161*_MUT_ = five of six *v GPR161*_WT_ = zero of 220; *P* = 8.8×10^−8^, Fisher’s exact test), which suggests that 1q cnLOH is a hallmark for *GPR161* deficiency and a molecular marker for the identification of patients with *GPR161* mutations.

## DISCUSSION

Recognition of tumor predisposition syndromes helps to optimize tumor surveillance programs and to improve clinical outcome. Recent progress in high-throughput genomics and access to larger patient cohorts enables the identification of novel tumor predisposition syndromes. In the current study, we have identified a novel tumor predisposition syndrome with SHH-activated and early-onset MB as the primary clinical manifestation. Carriers of a protein-truncating germline mutation in *GPR161* reported here developed MB_SHH_ before the age of 3 years. Overall, we estimate that *GPR161* mutations are responsible for approximately 5% of infant MB_SHH_ cases. All *GPR161* mutations that were identified within our patients with MB_SHH_ were also found to be present at a low carrier frequency in the general population (approximately one in 42,000 to one in 125,000 individuals), and analysis of two available parent-offspring trios (M20769 and MB13_03) demonstrated parental transmission in both affected patients. Of note, the frequency of germline *GPR161* protein-truncating variants in the general population is six in 10,000 individuals, and extrapolated across the global population one would anticipate approximately four million people worldwide to be affected (Data Supplement).

Patients with truncating germline *GPR161* mutations (genomic location: 1q24.2) showed recurrent LOH of *GPR161* as a second hit in the tumor. This primarily resulted from segmental cnLOH of 1q, an event that is completely absent among *GPR161* wild-type MB_SHH_ and can thus be considered a hallmark characteristic of *GPR161*-associated MB. cnLOH and the unmasking of germline mutations have previously been shown to be of importance in the inactivation of tumor suppressor genes and are described in a variety of different tumor types.^33^ The 1q cnLOH event most likely originates from mitotic recombination as a result of DNA double-strand breaks that necessitate the second, homologous chromosome as a template for DNA repair. Such double-strand breaks tend to be more frequent at common fragile sites, which are prone to breakage upon DNA replication stress.^34^ A common fragile site (FRA1F) is also present at the 1q cnLOH breakpoints in *GPR161*-associated MB (1q21.1 to 1q21.3). Moreover, the lack of 1q loss events among *GPR161* wild-type MB_SHH_ tumors and the preferential mode of *GPR161* inactivation, either focal or via cnLOH, suggests that 1q harbors dose-sensitive genes that might restrict MB_SHH_ tumor development. Future studies could therefore explore whether genes on 1q might serve as a substrate for novel treatment options based on synthetic lethal interactions. Of note, analysis of somatic mutations in known and putative MB driver genes revealed biallelic inactivation of *GPR161* as the sole shared event across the six patients, indicating that loss of *GPR161* is sufficient for tumor growth, which is consistent with results of recent *Gpr161* knockout studies in mice.^35^

GPR161 function is essential for several aspects of embryonic development and, among other things, is also relevant for granule cell (GC) proliferation.^36-38^ GCs of the cerebellum are by far the most numerous neurons in the brain and MB_SHH_ originates from their progenitors (GC progenitors [GCPs]).^39^ Proliferation of GCPs is critically regulated by the mitogenic activity of the SHH ligand. SHH secretion from cerebellar Purkinje cells at late stages of embryonic development boosts the postnatal mitotic rate of GCPs before differentiated cells exit the cell cycle to become postmitotic neurons.^40^ Consequently, constitutive activation of SHH signaling increases the proliferation rate and neoplastic transformation of GCPs.^41^ As Gpr161 acts as a negative regulator of the SHH pathway^37^ and prevents GCP overproduction, a loss of GPR161 activity is consistent with MB pathogenesis, which was recently demonstrated in a mouse model with a neural stem cell–specific *Gpr161* deletion.^35^ The timing of the GPR161–SHH interplay at the stage of GPC differentiation is in line with a vulnerable phase for MB development in early childhood. Similar to *PTCH1*- and *SUFU*-associated MB_SHH_ in Gorlin syndrome, patients in this study were typically diagnosed with MB during infancy.

In our study, patients were mainly children and other tumors could potentially occur at later stages of life. The oldest patient of the study (index patient, M20769, age 29 years) developed recurrent BCCs and a meningioma at age 24 in the radiation field. She also developed multiple hyperplastic GI polyps and a tubular adenoma with low-grade dysplasia. Her father, likewise a carrier of the *GPR161* germline mutation, has died of an adenocarcinoma of the colon at age 55 years. In a second family (SJMB335), both the maternal aunt and grandmother had ovarian cancer, but were not available for additional genetic testing. Other potential manifestations of *GPR161*-associated tumors are clearly an avenue for additional studies, not least because of their fundamental importance for genetic screening programs. In summary, the *GPR161* disorder defines a novel MB predisposition syndrome.
